# Cathodic-controlled and near-infrared organic upconverter for local blood vessels mapping

**DOI:** 10.1038/srep32324

**Published:** 2016-08-31

**Authors:** Chih-Hsien Yuan, Chih-Chien Lee, Chun-Fu Liu, Yun-Hsuan Lin, Wei-Cheng Su, Shao-Yu Lin, Kuan-Ting Chen, Yan-De Li, Wen-Chang Chang, Ya-Ze Li, Tsung-Hao Su, Yu-Hsuan Liu, Shun-Wei Liu

**Affiliations:** 1 Department of Electronic Engineering, National Taiwan University of Science and Technology, Taipei 10607, Taiwan; 2 Chang Gung University College of Medicine, Taoyuan City 33302, Taiwan; 3 Department of Ophthalmology, Chang Gung Memorial Hospital, Keelung City 20401, Taiwan; 4 Department of Electronic Engineering, Ming Chi University of Technology, New Taipei City 24301, Taiwan

## Abstract

Organic materials are used in novel optoelectronic devices because of the ease and high compatibility of their fabrication processes. Here, we demonstrate a low-driving-voltage cathodic-controlled organic upconverter with a mapping application that converts near-infrared images to produce images of visible blood vessels. The proposed upconverter has a multilayer structure consisting of a photosensitive charge-generation layer (CGL) and a phosphorescent organic light-emitting diode (OLED) for producing clear images with a high resolution of 600 dots per inch. In this study, temperature-dependent electrical characterization was performed to analyze the interfacial modification of the cathodic-controlled upconverter. The result shows that the upconverter demonstrated a high conversion efficiency of 3.46% because of reduction in the injection barrier height at the interface between the CGL and the OLED.

Among functional organic optoelectronics, upconverters have attracted considerable attention because of their potential applications in inspections, security, night vision and biomedicine^1^,^2^,^3^,^4^,^5^. Via integration of absorption and emitting components, a near-infrared (NIR) image can be converted into a visible pattern, which enables direct observation by the naked eye. For example, Kim *et al*. reported that a low-cost IR imaging camera with integration of a sensitizing layer (PbS nanocrystal or tin phthalocyanine:C_60_), a transparent phosphorescent organic light-emitting diode (OLED), and a digital single-lens reflex module was used to capture the pixel-less imaging in a dark environment with a 1.2 μm flash light^6^. Although the image-converted concept has been proposed in the literature^7^,^8^, previous studies have focused on the improvement in conversion efficiency, primarily because of the immature device optimization. The device structure of upconverters can be divided into three types: all-inorganic, hybrid, and organic upconverters. Because of the mature fabrication processes, inorganic-based upconverters, containing photodetectors (III-V compound semiconductor) and light-emitting diodes (LEDs), have been widely studied^9^,^10^,^11^. However, crucial issues remain on the heterogeneous lattice mismatch, which leads to poor efficiency and complicates the fabrication process.

Another study was, which followed up with the development of OLEDs, a hybrid system with an architecture containing OLEDs assembled on inorganic semiconductors^12^,^13^,^14^,^15^,^16^,^17^,^18^. The major concept depends on the fabrication process which deposits organic layers directly on to inorganic devices. Despite the flexibility of the process, amorphous organic layers can solve the lattice-mismatch issue, an additional carrier-injection layer inserted at inorganic/organic interface is required. In 2012, Chen *et al*. published a hybrid upconverter, which achieved an infrared-image conversion for the first time^8^. They pointed out that the interface between the carrier-generated InGaAs and OLED seriously affects the performance of upconverters. However, the low conversion efficiency of 0.57% and lateral carrier diffusion led to an inferior resolution of the image. To obtain a high-quality image in the upconverter, the problem of the lateral carrier spreading in the hybrid system needs to be suppressed. More recently, Kim *et al*. reported low-cost hybrid upconverters with a colloidal PbSe nanocrystal sensitizing layer, while a maximum photon (1.3 μm) -to-photon (0.52 μm) conversion efficiency approaching 1.3% has been achieved^15^. This work showed that the IR absorption range of upconverters can be extended by up to 1.5 μm as similar to an all-inorganic upconverter^11^, which may be applicable to the field of night vision.

On the other hand, for the development of photon-to-photon conversion efficiency, the So group published an efficient organic upconverter consisting of SnPc:C_60_ NIR sensitizer and phosphorescent OLED^19^, which reported the maximum conversion efficiency of 2.7% at a voltage of 15 V. Most importantly, the device configuration of the upconverter is very similar to a conventional OLED, resulting in the large-area device possibly being prepared via thermal evaporation processes. Based on the high compatibility of the fabrication process in thermal evaporation, the organic upconverter seems to be a promising alternative for traditional upconverters because of the low cost and simplified fabrication process^20^,^21^. To improve the efficiency of upconverters, taking as reference the tandem device of OLEDs may be an appropriate concept. The basic principle of the tandem device is that charges separated from hole-electron pairs will be generated inside the devices and transported in the opposite direction to their respective electrode. Therefore, a significant improvement of current efficiency in tandem OLEDs was achieved^22^,^23^,^24^,^25^. Based on a similar mechanism, a tandem upconverter with a photosensitive charge-generation layer (CGL) can be a new type of upconverter and thereby high conversion efficiency is expected. However, to develop tandem upconverters, the electron-supply mechanism should be demonstrated first because most of the studies focused on conversion efficiency and fabricated upconverters with hole-supply configurations. The applications of upconverters lie in the sequence of deposition processes and carrier-supply types, where upconverters with photosensitive-CGLs are inserted between the cathode and the OLED. Such device configuration is defined as cathodic-controlled device, which is still rare reported in this field.

In 2011, Okawa *et al*. was the only study to report on an upconverter with an electron-generation layer^26^. In addition to CGL, which was a blend of titanyl phthalocyanine (TiOPc) and C_60_, the upconverter had a bis[N-(1-naphthyl)-N-phenyl]benzidine buffer layer and an Al electrode. When the upconverter was illuminated by a 633-nm He-Ne laser, the device showed various current density-voltage (*J*-*V*) characteristics^27^. In addition, the maximum brightness only reached 200 cd/m^2^ at a high current density of 100 mA/cm^2^. Although the device was assembled using a bulk heterostructure sensitizer of TiOPc:C_60_, the upconverter exhibiting the 10 cd/m^2^ is required as a high-driving voltage of over 20 V. A possible reason for the high operation voltage of such a device was the absence of a carrier-injection layer to limit electron injection. Thus far, no study has reported on NIR cathodic-controlled upconverters with a low drive voltage of below 10 V and a photon-to-photon conversion efficiency of over 3%; the device exhibited with an emission brightness of 100 cd/m^2^. The injection or transporting-barrier should be considered by examining the working principles and energy levels of organic materials. For instance, despite the existence of various CGL configurations in tandem devices, the charge-injection layers remain essential and considerably affect the device performance^28^,^29^,^30^. In addition, Chen *et al*. published a tandem-OLED structure with a bipolar CGL which is similar to the CGL of upconverters^31^. They used an interconnecting layer, consisting of a blend of zinc phthalocyanine and C_60_, and a thin LiF layer to improve electron injection. Electron-injection layers (EILs) may be crucial component of the photosensitive electron-generation layer in upconverters.

More recently, we reported the transparent organic upconverter integrating with a bulk heterostructure sensitizer of chloroaluminum phthalocyanine (ClAlPc):C_70_ and phosphorescent OLED for real three-dimensional (3D) object sensing that can convert NIR light into a green emission in a dark environment and under NIR illumination^32^. In addition, the conversion efficiency exceeded 6% at 7 V and the image resolution achieved 400 dots per inch (dpi). Note that such an organic upconverter’s performance is the highest value reported to date^6^,^8^,^14^,^15^,^19^. To realize a new IR imaging device, in this paper we describe a 3.46% conversion efficiency, cathodic-controlled, and NIR organic upconverter for 3D mapping applications, where our proposed imaging system included several components, i.e. phosphorescent OLED, organic photosensitive CGL, transparent electrode, lens module, and NIR LED, to convert the NIR photon to visible light. Note that our proposed device is more likely to be part of a device-based upconverter, which is completely different to a simplified light upconverter without the use of any external stimulus^33^,^34^. Figure 1a shows a schematic of the imaging concept of our proposed cathodic-controlled NIR upconverter in the dark environment and under NIR illumination. When a commercialized NIR LED was used to illuminate the real object, then a reflected NIR photon from the outside object would be collected by an optical lens. Because our upconverter has sensitivity to an NIR signal, a 3D image of a real object was possibly obtained by a naked eye or a digital camera. Regarding our proposed NIR upconverter’s imaging system, a clear image of the blood vessels of a human forearm with 600 dpi has been achieved. In addition, to investigate the effect of the electron injection efficiency in the proposed upconverter, a temperature-dependent electrical characterization was performed to determine the energy barrier height between the EIL and the electron-transporting layer (ETL).

## Results

### Device configuration of the proposed upconverters

**Figure 1:**
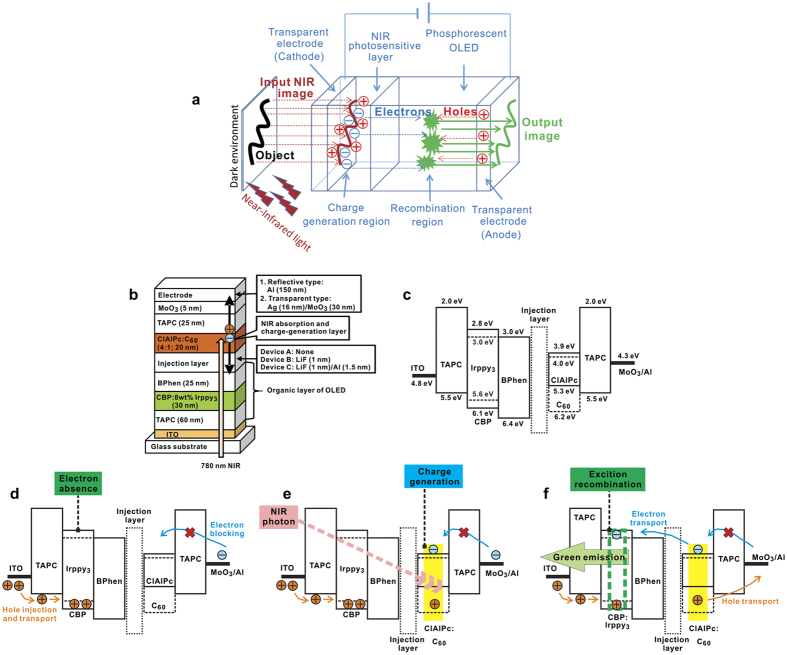
Device configuration, energy-level diagram, and operation mechanism of the proposed organic upconverters. (**a**) Schematic illustration of our proposed NIR imaging concept. (**b**) Device structure of the cathodic-controlled upconverters. (**c**) Energy-level diagram of materials used in the current study. Operation mechanism of organic upconverter when device was (**d**) in a dark environment (turned on) and (**e**) under NIR illumination to generate the hole and electron carriers via the excition generation and disassociation in the CGL. (**f**) Device emitted a green emission due to electron injection and carrier recombination in the EML.

The device structure of the proposed upconverter is shown in Fig. 1b. An indium-tin-oxide (ITO) pre-coated glass was used as substrates. A phosphorescent OLED was deposited layer-by-layer in sequence of 1,1-bis(di-4-tolylaminophenyl) cyclohexane (TAPC), 4,4’-Bis(N-carbazolyl)-1,1’-biphenyl (CBP) doped with fac-tris (2-phenylpyridine) iridium (III) [Ir(ppy)_3_], and 4,7-diphenyl-1,10-phenanthroline (BPhen), all of which were used as a hole-transporting layer (HTL), emitting layer (EML), and ETL, respectively. A blend layer, consisting of chloroaluminum phthalocyanine (ClAlPc) and C_60_ as a CGL, was deposited directly on the phosphorescent OLED, followed by a buffer layer TAPC, MoO_3_, and an Al cathode. Figure 1c shows an energy-level diagram of the upconverters. Figure 1d–f show the operating mechanism of upconverters. Because of the absence of electron injection from the cathode, the EML cannot emit visible photons, even when the device was under bias (Fig. 1d). When the device was illuminated by NIR irradiation, NIR photons can be absorbed by the CGL, and the free carriers will be generated via exciton-disassociation (Fig. 1e). Finally, electrons injected into the EML and the device emits visible green light (Fig. 1f). These steps were similar to those of the previous study with anodic-controlled upconverters^32^. Both exhibit the same behavior to perform the upconversion; devices absorb low-energy photons and generate carriers that recombine to emit high-energy photons. It is obvious that the barrier at the electron-transporting interface between the CGL and the BPhen layer is much higher than the hole barrier at the interface between the ClAlPc and TAPC layer. On the basis of the previous report, despite the fact that the CGL dominates the conversion efficiently and provides rich carriers, an EIL is still required. To investigate the electron-injection efficiency, a thin LiF (1 nm) layer was used in the experiments, as in the literature^31^. In addition, a thin layer LiF (1 nm)/Al (1.5 nm) was used for comparison. The upconverters without the EIL, with the LiF, and with the LiF/Al EIL were denoted as Device A, B, and C. A standard phosphorescent OLED with a structure of ITO/TAPC (60 nm)/CBP:8% Ir(ppy)_3_ (30 nm)/BPhen (25 nm)/LiF (1 nm)/Al (120 nm) was fabricated to be a reference. On the cathode side, an insertion of MoO_3_ layer was expected to block electron injection from the cathode into the TAPC layer. The optoelectrical characteristics were measured with a bottom-emitting device having a reflective cathode, Al (120 nm), in an atmosphere and in a dark room, without and with NIR illumination. The illumination source was an LED with a wavelength of 780 nm, exhibiting an average power density of 0.15 mW mm^−2^ over the entire active area (2 mm × 2 mm) of the upconverter. In addition, a transparent and large-area device (6 mm × 6 mm) was fabricated with a multi-layer transparent cathode, Ag (16 nm)/MoO_3_ (30 nm), and utilized to convert the NIR image via a focus lens. The transparent upconverter enables the observation of objects by the naked eye and a digital camera.

### Optical properties of materials used in this study

**Figure 2:**
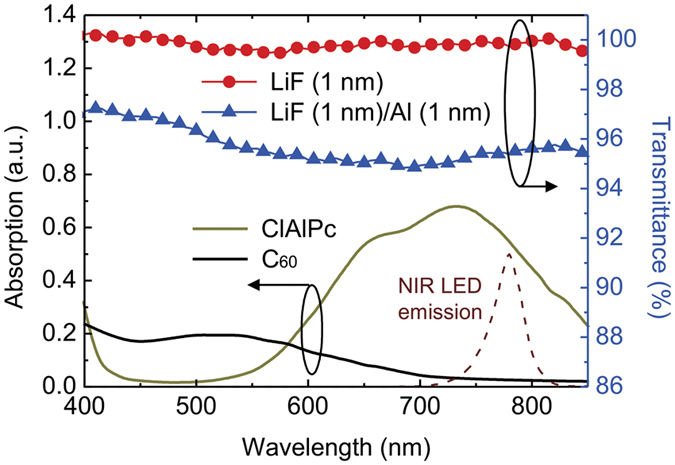
Optical properties of organic materials and the EILs. Absorption spectra of materials used in the CGL and transparency spectra of the EILs. Emission of the 780-nm NIR LED is provided for comparison.

The absorption spectra of the CGL are shown in Fig. 2. The ClAlPc with long-wavelength absorption properties, which covers the 780 nm of the NIR LED, was blended with C_60_ in a configuration similar to a bulk-hetrojunction organic photovoltaic device^35^,^36^. Since the EIL was inserted in the upconverters between the CGL and the OLED, the EIL may slightly block NIR illumination, thus reducing the NIR input. In addition, the insertion of the EIL may influence the light extraction of green emission emitted from OLED. Therefore, the optical influence of the transmittance on light output should be confirmed first. As shown in Fig. 2, by observing the region over 400 nm to 850 nm on transmittance, a thin LiF layer (1 nm) exhibited a transmittance of nearly 100%, while the LiF (1 nm)/Al (1.5 nm) layer showed a slightly decreased transmittance of approximately 96%. Nevertheless, the transmittance of the EIL is still high enough to avoid affecting the NIR input and the green output. In other words, the insertion of the EIL does not act as an embedded mirror to influence the optical properties, such as the microcavity effect, thus leading to an almost identical OLED emission and, hence, the conversion efficiency between the device with and without the EIL.

### Comparison of *J*-*V* characteristics of the proposed upconverters with various EILs in the dark and under 780-nm NIR illumination

**Figure 3:**
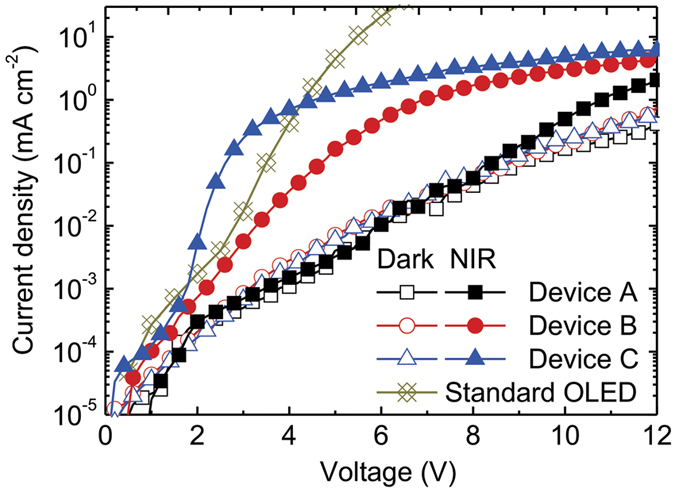
*J-V* characteristics of the upconverters with various EILs. The open and solid symbols denote the currents measured in the dark and under 780-nm NIR illumination of an average intensity of 0.15 mW mm^−2^, respectively. The characteristic of a standard OLED, as used in the upconverters, is provided. The *J*-*V* characteristics were measured using a bottom-emitting configuration with a thick cathode (i.e. NIR illumination and green emission on the same side).

Figure 3 shows the *J*-*V* characteristics of the upconverters with different EILs. Without NIR illumination, the dark currents of the three upconverters increased monotonically, and the values among the three were quite similar. The dark current leakage of the upconverters may be caused by charge carrier injection from two electrodes, for example the hole and electron carriers came from the anode and cathode, respectively. In this work, we used the buffer layer of TAPC/MoO_3_ between the upconverter and cathode to suppress the electron injection efficiency. Thus, this indicated that all electrons came from the NIR sensitizing layer when the device was applied with a forward bias. We observed that the currents among the three devices presented significant differences under NIR illumination. As expected, the NIR-induced current of Device A separated from the dark one was insignificant until the applied voltage reached 8 V. This result indicates the fact that the injection barrier between the CGL and the ETL of OLED results in a high driving voltage and low efficiency compared with other devices. Both Device B and Device C showed a considerable increase in the NIR-induced current when the voltage bias was over 2 V. Device C exhibited a more pronounced and dramatic increment in the NIR-induced current compared with Device B. In Device A without introducing the EIL, the energetic barrier present at the interface between the CGL and the ETL of OLED hindered the electron injection. When the EIL of the thin LiF was inserted, the injection barrier was reduced and the NIR-induced electrons could be injected into the ETL of the OLED. Furthermore, when the thin LiF/Al was used as the EIL, the electrons were injected into the ETL of the OLED more efficiently, thus leading to a dramatic increase in the NIR-induced current as observed from the kink at a voltage of approximately 2 V. All the upconverters exhibited the NIR-induced currents increased with the applied voltage and became saturated. This is on account of the fact that the maximum current density is limited by the carrier-supply amount provided by the CGL. For comparison, the standard phosphorescent OLED used in the current study is provided. Because the carriers of the OLED are primarily a result of the injection from both the anode and cathode, the current increased with the applied voltage. However, the kink, or the onset point of the standard OLED, was higher than the upconverter with the LiF/Al EIL, thus indicating that the upconverter can be turned on with a smaller driving voltage compared with the standard OLED.

### Emission properties as a function of applied biases of the proposed upconverters with various EILs

**Figure 4:**
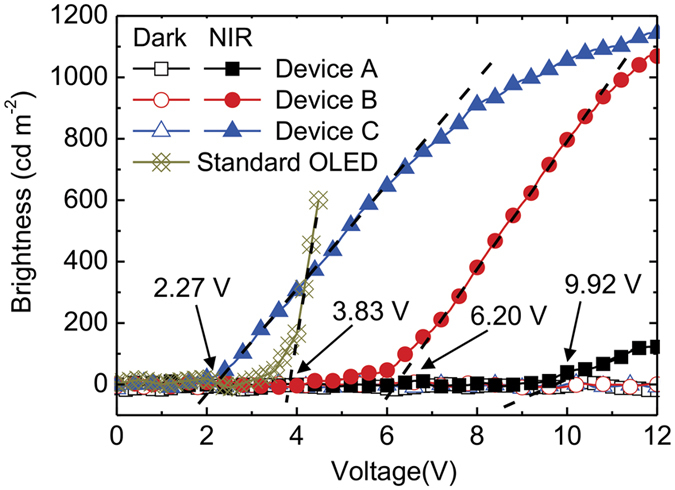
*B-V* characteristics of the upconverters with various EILs. The open and solid symbols denote the brightness measured in the dark and under 780-nm NIR illumination, respectively. The characteristic of the standard OLED as used in the upconverters is provided. The driving voltage of each device is pointed out by an arrow, together with text indicating the corresponding voltage. The *B*-*V* characteristics were measured using the bottom-emitting configuration with a thick cathode (i.e. NIR illumination and green emission on the same side).

To confirm the performance of the devices, the brightness-voltage (*B*-*V*) characteristics of the upconverters were measured, and are shown in Fig. 4. In the dark condition, the upconverters were supposed to be turned off due to only the hole current from the anode and a small amount of electron leakage current inside the devices, thus resulting in an extreme imbalance of the carriers for radiative recombination. Although the dark current increased with the applied voltage, as illustrated in Fig. 3, the devices do not emit a noticeable intensity of brightness which can be detected by a spectrophotometer. Because of the absence of an injection layer in Device A, the electrons generated from the CGL are unlikely to be injected into the ETL of the OLED, and so reasonably lead to low brightness. In Fig. 4, we can roughly estimate the onset point, which corresponds to the driving voltage, 9.92 V, 6.40 V and 2.27 V, for Devices A, B, and C, respectively. Device A exhibited the highest onset voltage and lower output brightness. Although there was a slightly poor electron injection in Device B, it achieved a high brightness of 1069 cd m^−2^ at 12 V, which is close to Device C with the brightness of 1144 cd m^−2^ at 12 V. Therefore, the LiF/Al EIL can considerably reduce the electron-injection barrier at the interface between the CGL and the ETL of OLED, thus providing the lowest driving voltage and the maximum brightness. The photon-to-photon conversion efficiency, *η*_CE_, was calculated by the following equation^6^,^19^:


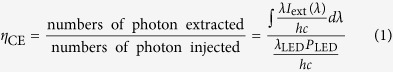


where *h*, *c*, *λ*, *I*_ext_(λ), *λ*_LED_, and *P*_LED_ are the Planck constant, the speed of light, the wavelength of photon, the external emitting intensity of the upconverter, the wavelength of the NIR LED, and the incident power of the NIR LED, respectively. The estimated conversion efficiency of Device C is 3.46%, which is the highest value reported to date. The proposed upconverter with the optimal EIL outperformed the previous upconverters with both the hole- and electron-supply configurations.

### Estimation of the interfacial barrier between the ETL of the OLED and the CGL with various EILs

**Figure 5:**
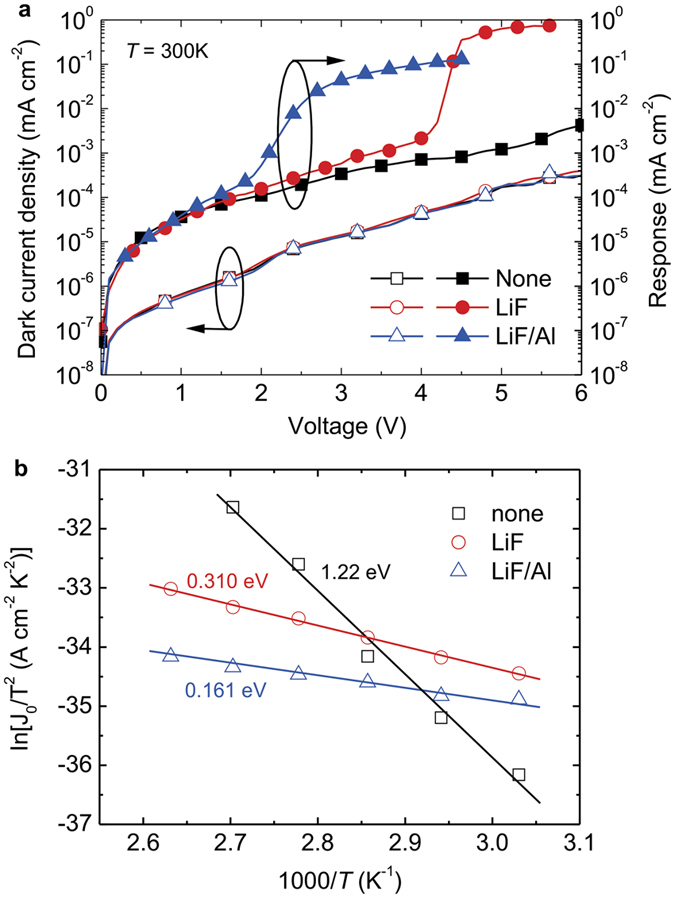
Electrical characterization for estimating the interfacial barrier between the CGL and the ETL of the OLED. (**a**) *J-V* characteristics of the electrical characterization for the devices with various EILs. The open symbols denote the currents measured in the dark, and the solid symbols show the responding current density as defined by the actual light current (under 780-nm NIR illumination of an average intensity of 0.15 mW mm^−2^) minus the dark current. (**b**) Relationship between ln(*J*_0_/*T*^2^) and (1000/*T*) for devices with various EILs. The solid lines are the fits according to Eq. 3. The text indicates the interfacial barrier.

To estimate the electron barrier between the CGL and ETL of the OLED, a device structure of ITO/BPhen (100 nm)/EIL/ClAlPc:C_60_ (4:1; 20 nm)/BPhen (10 nm)/Al (120 nm) was fabricated and a temperature-dependent measurement was performed. Because of a wide band gap of the BPhen, holes and electrons cannot enter the devices. When 780-nm NIR source illuminated on the devices, the carriers are generated in the CGL. By applying positive and negative bias on the ITO and Al electrode, respectively, holes and electrons transport to their respective electrode. In an ideal case, holes are blocked by the BPhen near to the Al because of the energetic barrier, while electrons are injected into the BPhen near to the ITO and contribute to the output current. Unlike the previous reports, which normally investigated the energetic barrier between organic materials and electrodes, a device structure and measurement are proposed to estimate the energetic barrier at the interface between the organic layers, the CGL and the ETL of the OLED. Figure 5a shows the results of dark and responding current density for different devices with various EILs. The *J*-*V* characteristics were similar to those of the upconverters, thus exhibiting small dark currents and considerably higher NIR-induced currents. The low dark current density was a significant proof of the carrier block at either anodic or cathodic interface, although the small leakage currents increased monotonically with the applied voltage. Based on the theory of thermionic emission current, we can calculate the energetic barrier using the following the equation^37^,^38^:


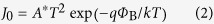


where *J*_0_, A^*^, *q*, *k*, *T*, 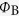, are the field-free current density, Richardson constant, the elementary charge, Boltzmann constant, the temperature, and the interfacial barrier, respectively. The *J*_0_ was deduced from the linear relations between ln*J* and (*V*−*V*_bi_)^1/2^, as shown in Fig. S1a–c, where *V*_bi_ is the built-in potential in the device and assumed to be 0.5 V, because of the work function difference between the ITO (4.8 eV) and the Al (4.3 eV). By plotting the ln(*J*_0_/T^2^) against (1000/*T*), the interfacial barrier can be deduced from the slope of the curve. Figure 5b shows the fitting results. The interfacial barrier was 1.220, 0.310, and 0.161 eV, for the devices without the EIL, with the LiF EIL, with the LiF/Al EIL, respectively. This result indicates that the interfacial barrier can be reduced successfully by inserting the EIL in the electron-supply upconverters, resulting in a device with high current density under NIR illumination. In addition, the driving voltage of the device may be limited by the amount of carriers generated in the CGL. To confirm such an assumption, we can compare the performances of current density and brightness in Device C and a standard OLED (see Figs 3 and 4). The results show that the electrons generated from the CGL are more efficient in improving the driving voltage than in the standard OLED. The reduced barrier using LiF/Al EIL presented not only the low driving voltage but also the charge balance in the OLED^19^, thus leading to the highest conversion efficiency. Therefore, the insertion of an optimal EIL considerably reduced the electron-injection barrier and contributed to an efficient hole-electron recombination in the EL and, hence, the promising optical and electrical properties in the device.

### Demonstration of the proposed upconverter in the dark and under 780-nm NIR illumination: NIR upconverter for local blood vessel mapping application

**Figure 6:**
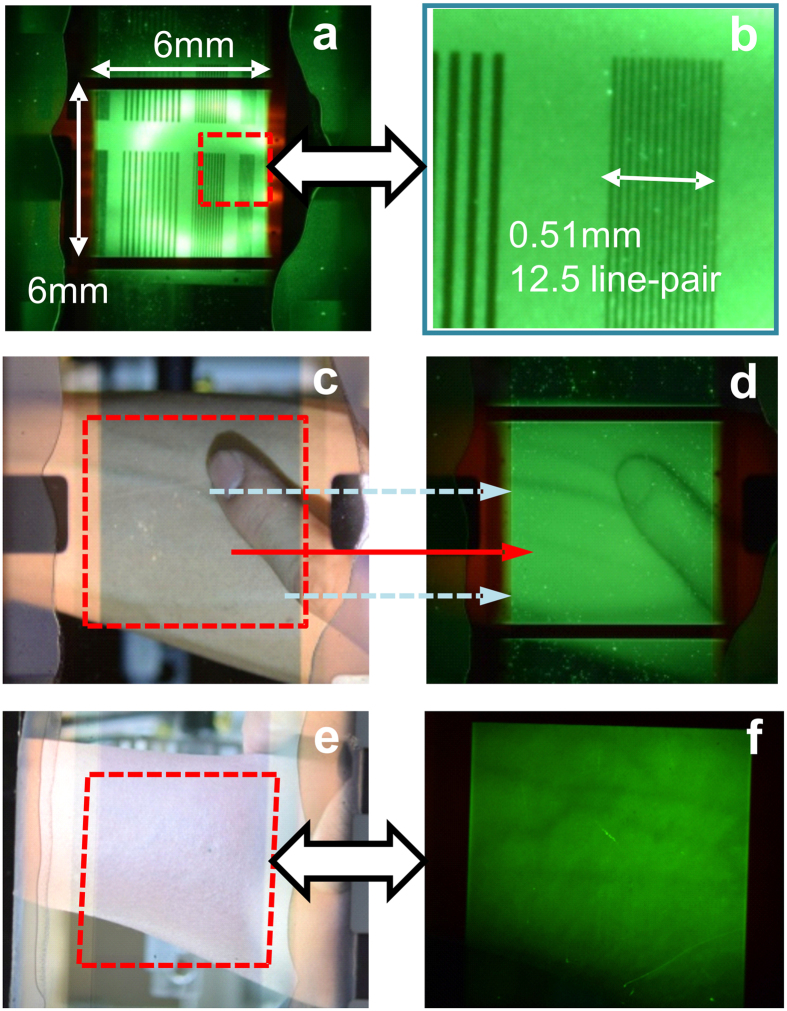
NIR upconverter for local blood vessel mapping application. (**a**) Real converted image of the line-shaped shadow mask captured by the transparent upconverter under NIR illumination. (**b**) Zoom-in image from the area marked by the dash square. (**c**) Observing human forearm and finger through the transparent upconverter captured by a digital camera. (**d**) Converted NIR image captured by the transparent upconverter in a dark environment. (**e**) Human forearm shows a smooth morphology and insignificant vein shape. (**f**) Converted NIR image shows vein position with a dark brown color captured by the transparent upconverter in a dark environment. NIR illumination and OLED emission passed through the ITO glass substrate and transparent cathode, respectively.

To demonstrate the NIR-mapping application, a transparent upconverter with a large active area (6 mm × 6 mm) and a transparent electrode (Ag (16 nm)/MoO_3_ (30 nm)) was fabricated. This device structure enables obtaining a clear image by applied the constant voltage of 5 V and under NIR illumination; objects are brought into focus by using a focus lens. The system setup is shown in Fig. S2a. The line-shaped shadow mask was clearly observed when the object was illuminated by a 780-nm NIR LED in darkness. Figure 6a shows a magnified view of the region marked by a dashed square. As shown in Fig. 6b, the line-pairs were clearly distinguishable. The image quality was investigated by considering the resolution of the image. The calculated method was reported in a previous paper^32^, where the 12.5 line pairs lie within 0.51 mm, implying a maximum image resolution of 600 dpi. The high dpi suggests that the thin LiF/Al layer inserted into the upconverter does not cause lateral current spreading. Moreover, the layer not only reduces the injection barrier height but also ensures a high-quality image. NIR image sensing technology has been developed for application of biometric identification, such as capturing the vein print of fingers. Currently, to capture an NIR image, a complex system consisting of a photosensitive device, printed circuit board, and display monitor is required. In this study, we used a cathodic-controlled upconverter for imaging local blood vessels and demonstrated its practicality. Because of the transparency of biological tissues and the high absorption of NIR by veins^39^, photons with a wavelength of 780 nm can penetrate human skin by a few millimeters and was possibly used to image the shape of blood vessels (90% of the blood and 10% of the vessel wall)^40^. Figure 6c,e present a view of a forearm through the transparent upconverter under normal room lighting; a clear image of the forearm is visible. When the lighting source was switched to NIR illumination in darkness, the veins were darker in color. Hence, their precise position under the skin could be determined directly from the green image on the upconverter, as shown in Fig. 6d,f. In Fig. 6c, the solid lines represent specific blood vessels in the human forearm (see Fig. 6d), and the dashed line denotes an obscure blood vessel located deeper below the skin. A careful examination of the difference in the images obtained with normal room lighting (fluorescent tube with white light, as shown in Fig. 6c,e) and NIR illumination (transformed by the upconverter into green color as shown in Fig. 6d,f) shows that some details of the skin. Despite the limited information obtained under various conditions, the main contributions of our upconverter are the direct observation of veins in a different color and their surface status in NIR images. The forearm presented in Fig. 6e shows a deep skin color and obscure blood vessels. Discerning the blood vessels under room lighting was difficult, even by applying pressure on the elbow. Unexpectedly, the shape of the blood vessels was obtained accurately by our proposed upconverter’s system due to the high absorption of NIR by veins to form the dark pattern, as shown in Fig. 6f. Note that such experiment was approved by the Institutional Review Board of Chang Gung Memorial Hospital, Taiwan (see “Methods” section). The proposed transparent upconverter facilitates nondestructive sensing of blood vessels, which cannot be achieved using inorganic photosensitive devices, such as charge coupled devices or complementary metal-oxide-semiconductors.

## Discussion

Conventional upconverters based on inorganic or organic materials as CGLs have been fabricated recently. Therefore, previous studies have primarily used CGLs to provide holes and have focused on the hole-injection properties. However, the conversion efficiency of an upconverter is still too low. For increasing the conversion efficiency, the use of a tandem structure involving two or more upconverters is a promising approach to achieving higher device performance. The development of an ambipolar CGL is necessary for connecting OLEDs at the top and bottom. Based on our research, no previous study has attempted to fabricate an organic tandem upconverter. A possible reason is attributed to poor electron injection efficiency in CGL to limit device performance. For example, Okawa *et al*. is the only study to report on the electron injection mechanism in CGLs^26^. Although a photoresponse was achieved, no other demonstrated a realistic application such as imaging and calculation of quantum efficiency. In the present study, we demonstrated a CGL that enabled generating electrons and an EIL that activated electron injection to an OLED for emitting light. The upconverter showed poor photoresponse and emission in the absence of the EIL. When a thin LiF layer was inserted between the ETL of the OLED and the CGL, the turn-on voltage and device performance improved compared with the device without an EIL. A considerable improvement was achieved when the LiF deposition was followed by the deposition of a thin Al layer. Although the LiF/Al layer was inserted between the OLED and the CGL, the total thickness was 2.5 nm, which slightly reduced the transparency to approximately 96%. Therefore, either the NIR light input into the CGL or the light output from the OLED was more relevant to the insertion of the LiF/Al EIL. In addition, numerous electrons can be injected into the ETL of the OLED for recombination with holes injected from the anode. Consequently, an extremely low driving voltage and high brightness under 780-nm NIR illumination can be achieved. The calculated external quantum efficiency was approximately 3.46%, which is the highest value reported to date. Although this value is low compared with that of commercial instruments, we demonstrated the feasibility of obtaining NIR derived images. The NIR image of a line-shaped shadow mask showed that the proposed upconverter can produce images with a resolution of 600 dpi. We also integrated the organic upconverter with a commercial digital camera to demonstrate a novel application: local blood vessel mapping. The location and shape of the blood vessels of a human forearm were clearly observed in the NIR images. We believe that this research will prompt additional research in this field and promote the application of organic upconverters.

In summary, we demonstrated an organic cathodic-controlled upconverter exhibiting high photon-photon conversion efficiency of 3.46% by using a photosensitive CGL. The experimental results showed that the insertion of an EIL affected the upconverter characteristics substantially. By optimizing the EIL, an upconverter with a low-driving voltage of approximately 2 V was achieved. A temperature-dependent electrical characterization was performed to describe and calculate the electron injection barrier comprehensively, thereby favoring the design principles of the electron-supply upconverters. In addition, on integration with a commercial camera-lens focus, the transparent single-pixel upconverter with a large-area (6 mm × 6 mm) could convert an NIR image into a visible green image with a resolution considerably higher than 600 dpi. Thus, the applicability of the organic upconverter as an NIR imaging device for local blood vessel mapping was demonstrated.

## Methods

All materials, TAPC, CBP, Ir(ppy)_3_, BPhen, ClAlPc, C_60_, LiF, MoO_3_, and Al were purchased from Sigma-Aldrich. Glass substrates with pre-coated ITO having a sheet resistance of approximately 15 Ω/sq were purchased from Luminescence Technology Corporation. Prior to thin-film deposition, the ITO substrates were soaked in detergent, DI water, isopropyl alcohol, and acetone in an ultrasonic bath for 5 minutes sequentially. Then, the substrates were treated with high power oxygen plasma (150 W) for 5 minutes to modify the workfunction of ~5.6 eV^41^. Note that the ITO substrate without any plasma treatment was used for preparing the temperature-dependent device. All thin films were deposited in a high vacuum chamber with a pressure lower than 8 × 10^−6^ Torr. The device active area was defined by depositing the cathode through shadow masks with different widths, 2 mm and 6 mm, to fabricate the devices for performance characterization and NIR-image demonstration. After the cathode deposition, the devices were delivered to a nitrogen-filled glove box and encapsulated appropriately with a covered glass and an UV-curable epoxy resin (Everwide EXC345). The *B*-*J*-*V* characteristics were measured with a source meter (Keithley 2636A) and a spectrophotometer (Photoresearch PR-655). Temperature-dependent electrical characterization was carried out in a cooled cryostat (Janis VPF-100), equipped with a cryogenic temperature controller (Lake Shore 335) and a liquid-nitrogen cooling system. A 780-nm wavelength LED (Thoralbs LED780E) was used as an illumination source, which was determined by a power meter (Newport Model 1918-R) to measure the average power density of 0.15 mWmm^−2^ on the upconverter’s surface. A commercial digital camera (Nikon D5300) was used to capture NIR-converted images on transparent upconverters. This study was approved by the Institutional Review Board (IRB) of Chang Gung Memorial Hospital, Taiwan (IRB No. 201600317B0) in 2016. Informed consent was obtained from all subjects. The procedures used conformed to the tenets of the Declaration of Helsinki. The IRB certification was attached in the supplementary information.

## Additional Information

**How to cite this article**: Yuan, C.-H. *et al*. Cathodic-controlled and near-infrared organic upconverter for local blood vessels mapping. *Sci. Rep.* **6**, 32324; doi: 10.1038/srep32324 (2016).

## Supplementary Material

Supplementary Information
