# Supplementary material for: Cathodic-controlled and near-infrared organic upconverter for local blood vessels mapping
Source: Sci Rep. 2016 Aug 31;6:32324. doi: 10.1038/srep32324 (PMC5006079; doi:10.1038/srep32324)

Supplementary Information

Cathodic-controlled and near-infrared organic upconverter for local blood vessels mapping

Chih-Hsien Yuan^1^, Chih-Chien Lee^1*^, Chun-Fu Liu^2,3^, Yun-Hsuan Lin^2,3^, Wei-Cheng Su^1^, Shao-Yu Lin^1^, Kuan-Ting Chen^1^, Yan-De Li^4^, Wen-Chang Chang^1^, Ya-Ze Li^1,4^, Tsung-Hao Su^4^, Yu-Hsuan Liu^1^ & Shun-Wei Liu^4*^

Correspondence and requests for materials should be addressed to: [cclee@mail.ntust.edu.tw](mailto:cclee@mail.ntust.edu.tw) (C.-C. Lee) and [swliu@mail.mcut.edu.tw](mailto:swliu@mail.mcut.edu.tw) (S.-W. Liu)

^1^Department of Electronic Engineering, National Taiwan University of Science and Technology, Taipei 10607, Taiwan

^2^Chang Gung University College of Medicine, Taoyuan City 33302, Taiwan

^3^Department of Ophthalmology, Chang Gung Memorial Hospital, Keelung City 20401, Taiwan

^4^Department of Electronic Engineering, Ming Chi University of Technology, New Taipei City 24301, Taiwan

**S1. Temperature-Dependent Measurements**:

The current density in devices can be expressed by the following equation proposed by Matsumura *et al.*:

$J=J_{0}\exp\left(\left\{q\left[q\left(V-V_{\text{bi}}\right)/4\pi\varepsilon\varepsilon_{0}d\right]^{1/2}\right\}/kT\right)$ (S1)

where *J*_0_, *q*, *T*, *V*_bi_, *ε*, *ε*_0_, *d*, and k are the field-free current density, the elementary charge, the temperature, the built-in voltage caused by the difference of work functions between the anode and the cathode, the relative dielectric constant of the medium, the permittivity of free space, and the thickness of the transporting layer, respectively. Fig. S1 shows the relationship between ln*J* and (*V*-*V*_bi_)^1/2^ of the devices with a structure of ITO/BPhen (100 nm)/EIL/ClAlPc:C_60_ (4:1; 20 nm)/BPhen (10 nm)/Al (120 nm). The results for the devices without the EIL, with the LiF (1 nm) EIL, and with the LiF (1 nm)/Al (1.5 nm) EIL correspond to Figs. S1A-C, respectively. The field-free current density *J*_0_ can be obtained from the intercept in the figures. The consistently linearly proportional relation indicates that the current flow inside the devices is dominated by the thermionic emission.

**Fig. S1**. **│** **Interfacial barrier between the CGL and the ETL of the OLED.** The relationship between ln*J* and (*V-V*_bi_)^1/2^ of the devices: (**a**) without the EIL (denoted as none), (**b**) with the LiF (1 nm), and (**c**) with the LiF (1 nm)/Al (1.5 nm) EIL.

**S2. Setup of Image-Sensing System**:

To demonstrate a NIR image conversion, the system schema is shown in Fig. S2A. For image sensing, an object lens (Sony DT 1.8/35 SAM) was used. A commercial digital camera (Nikon D5300) was used to capture converted images on the upconverters. Fig. S2B shows a metal shadow mask with pre-designed line shape which was used to present the image quality. A cluster of LEDs (Fig. S2C) provided more uniform illumination than that of a single LED. The NIR lighting source was illuminated on real objects, such as shadow mask and human forearm. The reflecting photons were firstly collected by the object lens, and then focused upon the upconverters to form an image.


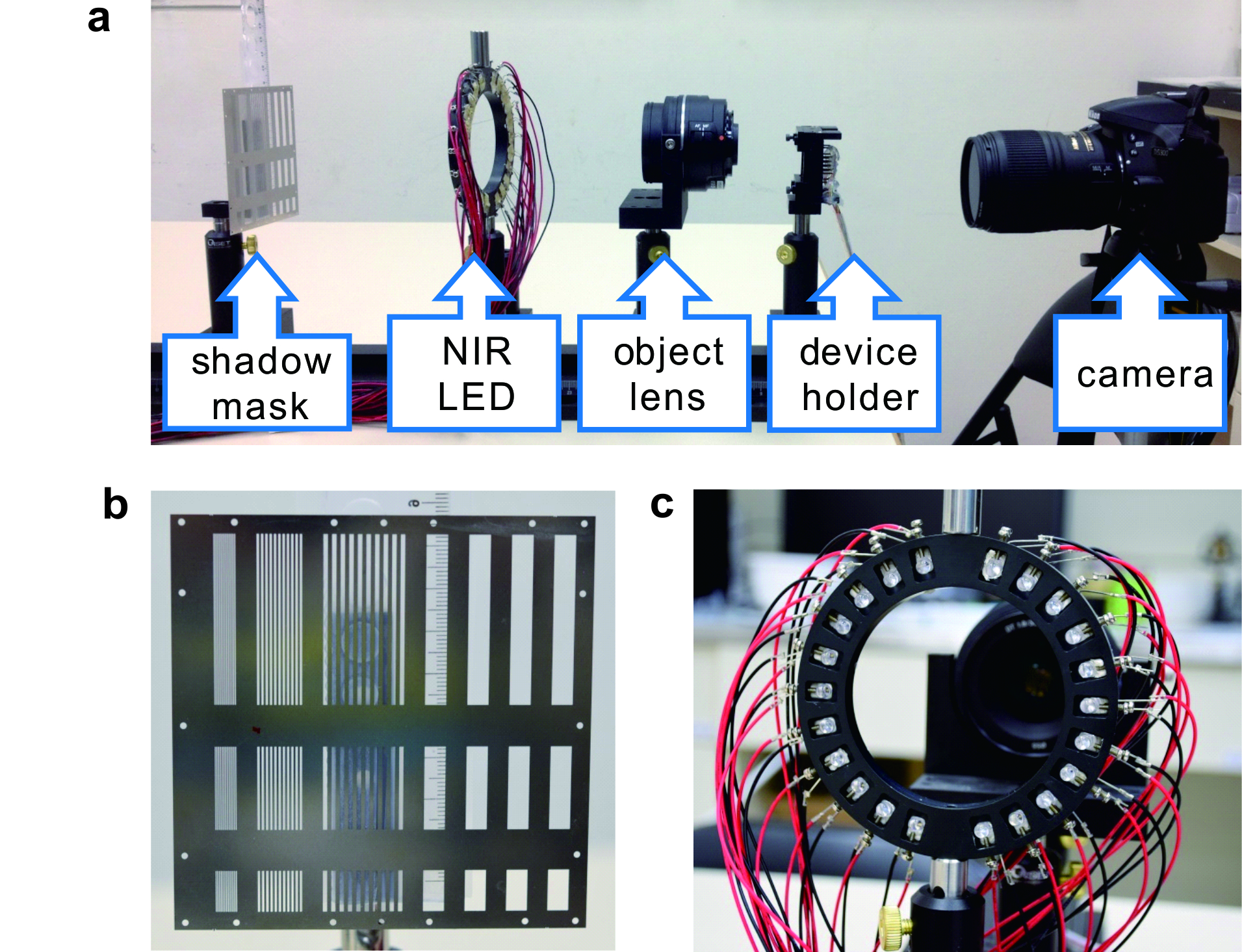


**Fig. S2**. **│** **Image-sensing system and components.** (**a**) Schema of the image-sensing system. (**b**) Picture of the line-shaped shadow mask for the resolution checking. (**c**) Picture of the circular holder bonding with twenty-two 780-nm LEDs.

**S3. Chang Gung Medical Foundation for Institutional Review Board**:


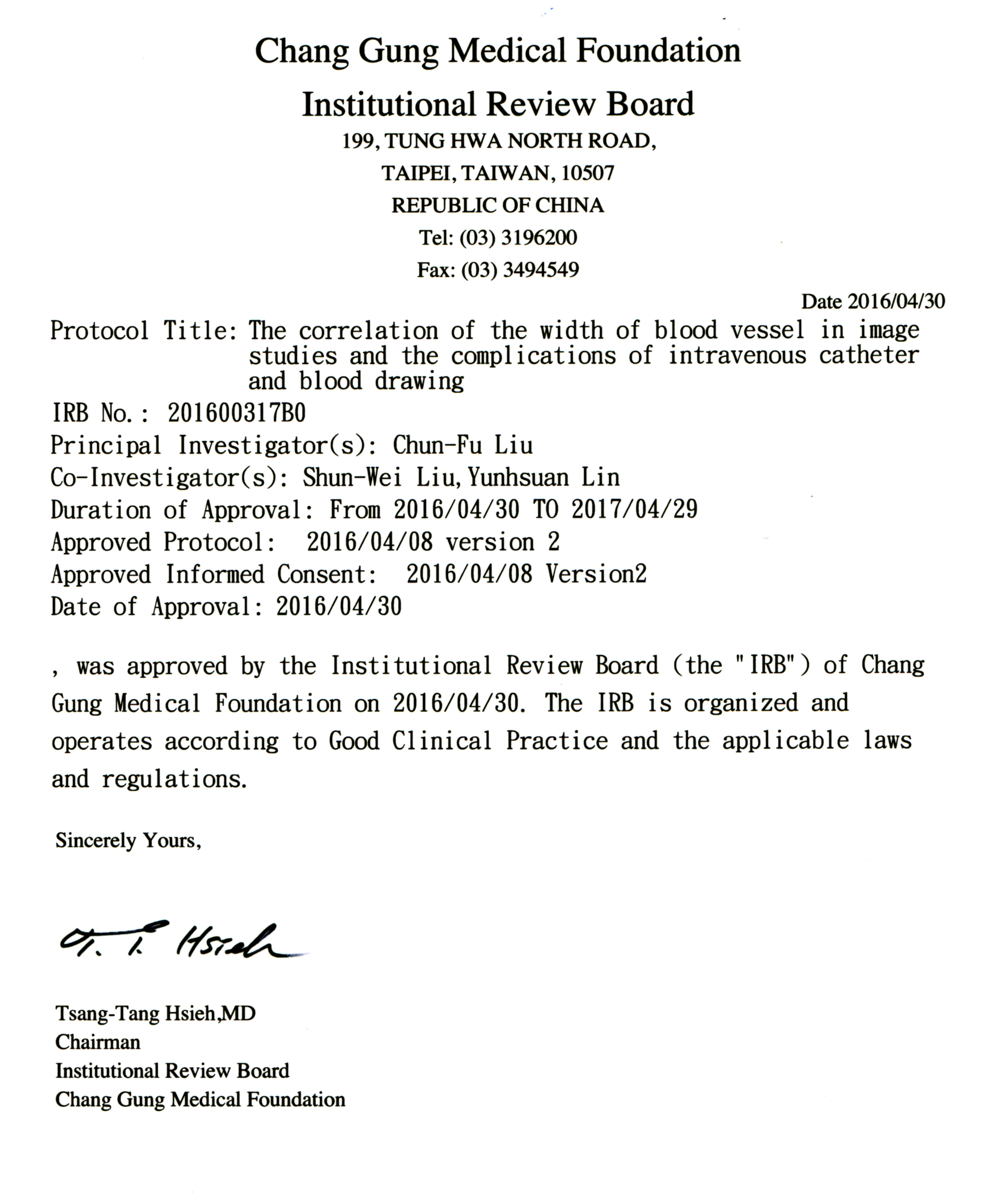


**S4. The committee for Institutional Review Board**:


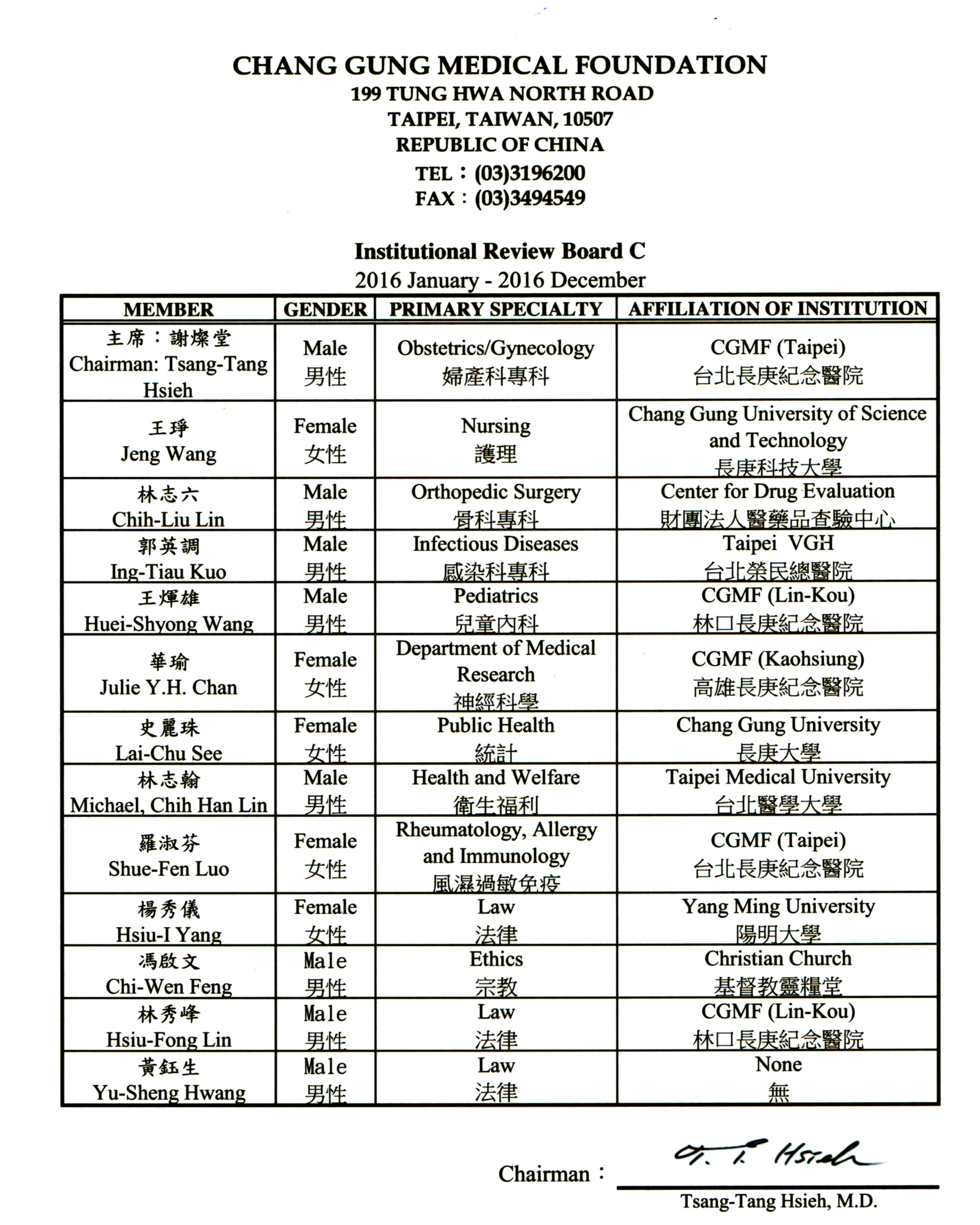

Supplement: Supplementary Information [file srep32324-s1.doc]
